# Report on the second Intracranial Hypertension Research Foundation conference

**DOI:** 10.1186/1743-8454-5-13

**Published:** 2008-08-13

**Authors:** Emanuel Tanne

**Affiliations:** 1 Intracranial Hypertension Research Foundation, 6517 Buena Vista Drive, Vancouver, WA 98661, USA

## Abstract

This report highlights a conference designed for patient education on elevated cerebrospinal fluid (CSF) pressure. The conference centered on chronic intracranial hypertension (IH) including the latest research and clinical information. It was sponsored by the Intracranial Hypertension Research Foundation and held at the University of Texas Medical School, Houston, on June 21–22^nd^, 2008.

## Background

The Intracranial Hypertension Research Foundation (IHRF) in Vancouver, Washington, promotes research into the pathophysiological basis of chronic IH and the evolution of better treatment and, ultimately, a cure. IHRF is a multi-functional organization. It not only encourages research, but also facilitates understanding and management of chronic primary and secondary IH, through research, training and education programs worldwide.

IHRF sponsors programs for researchers and clinicians, as well as educational conferences for patients and families. The Houston conference was attended by 108 patients and family members who heard presentations by nine clinicians and researchers.

Patients asked many questions during extensive panel sessions. The conference also allowed patients to speak about personal experiences with the disorder. IHRF held the first conference on chronic IH in 2006 at the Oregon Health & Science University (OHSU). A wide variety of subjects were presented to enhance patient knowledge and awareness. Patient education importantly improves patient insight about the disorder. It also facilitates active cooperation with their physicians. Understanding limitations of accepted medical and surgical treatment leads to realistic goals in management.

Additionally, patient education is vital in controlling health care costs.

## Discussion

The opening presentation by Conrad Johanson, Professor of Clinical Neuroscience at Brown University dealt with CSF production in the choroid plexus and its possible role in IH. Covered in the lecture was the structure of the choroid plexus, the dynamic turnover of ions and water in CSF production by the choroidal epithelium, the transport of CSF solute for the brain, and homeostasis of CNS extracellular fluid. A discussion of translational research goals involving the choroid plexus and CSF and possible directions for research in IH concluded the presentation.

John McGregor, Associate Professor of Neurosurgery at Ohio State University, spoke on "Neural Hydrodynamics Disorders: Hydrocephalus, Intracranial Hypertension and the Chiari Malformation. Relationships, Similarities and Differences" which was followed by two lectures covering CSF shunt and valve technology. The latter two presentations discussed in depth the available technology with details of the features of each device.

The conclusion was that despite the numerous designs, the overall success rate for the various shunts and valve equipment are similar. Better designed shunts are greatly needed.

The next lecture by Conrad Johanson: "Current Theories on Causation and Reduction of Elevated CSF Pressures: Implications for Intracranial Hypertension", began with a discussion of CSF fluid dynamics centering on CSF reabsorption including the controversial arachnoid and CSF lymphatic drainage. The role of neuropeptides such as atrial natriuretic peptide (ANP) in CSF production and the role of growth factors like basic fibroblast growth factor (FGF2) in CSF reabsorption, were identified. The intriguing possibility of peptides as pharmacological agents in controlling intracranial pressure (ICP) was thought provoking and stimulating, especially since no specific drug is available to control production and egress of CSF.

Steven Katz, neuro-ophthalmologist and Associate Professor of Ophthalmology at Ohio State University, lectured on the symptoms and signs in idiopathic intracranial hypertension (IIH) and the medical management options. He discussed approaches that work best in his practice. Steven Katz had previously demonstrated the somatostatin receptors 1 and 2 in normal human choroid plexus and arachnoid granulations and thus surmised that somatostatin is involved in CSF production and egress. His preliminary clinical use of octreotide, a peptide that mimics somatostatin, was discussed, including the potential complications of somatosatin analogs to control IH. Steven Katz described his techniques for optic nerve sheath decompression and demonstrated findings from many of his procedures. He attributed excellent outcomes to reduced surgery duration, i.e. especially the time of optic nerve stretching. He emphasized that short exposure and optimal surgical approach lead to minimal diplopia, ptosis and most importantly, minimal vision loss.

Marc Criden, neuro-ophthalmologist and Assistant Professor of Ophthalmology at the University of Texas, Houston, presented an updated overview of pediatric IH. In children under 10 years, gender and weight are not the factors they are in adults. He pointed out that many physicians consider IH in children under 10 to be a different disorder because of these characteristic differences. Headaches associated with IH and how best to manage them were discussed by neurologist and neuro-ophthalmologist, Leonard Hershkowitz of Baylor University. He indicated his management techniques that worked well for him.

A reception followed in which patients again communicated with the speakers.

The 2nd conference day opened with a discussion of the mission and goals of IHRF and a review of IHRF-sponsored research by Emanuel Tanne, Clinical Assistant Professor of Ophthalmology, OHSU and president of IHRF. He pointed out that IHRF works to remove significant obstacles to research: under-funding, under-coordination of effort, incomplete recognition of the life-altering effects of IIH, and low recruitment of researchers in this area. IHRF funded animal model development projects at the University of Chicago and the University of Arkansas. Under investigation is a knockout mouse that develops IH shortly after weaning. Other research areas included joint testing at the University of Utah of a NASA-developed, non-invasive, closed loop ultrasound device to measure ICP in microgravity and an investigation of vitamin A receptors in arachnoid granulations at Ohio State University. IHRF also partners with the Casey Eye

Institute, in regard to the Intracranial Hypertension Registry at the Oregon Health & Science University, Portland. The IH Registry is a relational database management system designed for medical research. Emanuel Tanne discussed ongoing Registry research, including studies in genetics, economics, weight gain and pregnancy. Jessica Tanne, IHRF Director, Communications & Development, discussed raising community awareness, fundraising and the importance of becoming an IH ambassador. Illustrating and discussing their fundraising creativity were Dori Clements and Jacque Tate, both parents of IH children.

Clark Sitton, Assistant Professor of Radiology, University of Texas, Houston, discussed types of imaging studies and the goals of imaging in IH. He presented an extensive collection of studies demonstrating pathological findings associated with IH.

Bariatric surgery as a possible option in the management of IH was presented by Erik Wilson, Assistant Professor of Surgery, University of Texas, Houston, who covered risks and benefits of bariatric surgery, using his extensive experience as a guide. He also discussed types of procedures available and his personal approach to follow-up and long term goals for patients. Marc Criden presented a new hypothesis to consider for clinical management of chronic IH: establishing target pressures for each patient with IH. The hypothesis arose out of his and Steven Katz's experience with patients at the last IHRF patient conference, where they were impressed by the variety of symptoms and patient reports contradictory to current teaching. They hypothesized that chronically elevated ICP is neurologically damaging, even in patients without significant visual dysfunction or intractable headache. They are presently investigating and evaluating this hypothesis. The final speaker, Kapil Kapoor, resident in ophthalmology at the University of Texas, Galveston, presented his research on hyposmia and IH. He found that patients with elevated ICP have a decreased sense of smell and concluded that other nerves such as the olfactory nerve may be functionally compromised in the setting of IH by a mechanism similar to that of optic nerve compression. Therefore, it may be appropriate to consider IH as a more global neuroanatomic insult of augmented CSF pressure than previously considered.

## Conclusion

While the intent of educating patients was admirably accomplished by this conference, the conference served as a unique experience for researchers and clinicians to hear from a large group of patients about the nature of their disorders. Therefore, not only did physicians from a variety of sub-specialties have an opportunity to exchange ideas and explore future collaborative projects they also left with a different perspective of chronic IH and a new appreciation of patient-centered conferences as a mechanism to expedite translational CSF research.

## Competing interests

The author declares he has no competing interests.

## Authors' contributions

I am the sole author and have read and approved the final version of this paper.

